# Supplementary figures and images for: Long-read RNA sequencing of human and animal filarial parasites improves gene models and discovers operons
Source: PLoS Negl Trop Dis. 2020 Nov 16;14(11):e0008869. doi: 10.1371/journal.pntd.0008869 (PMC7704054; doi:10.1371/journal.pntd.0008869)

# *Dim-tax-4*

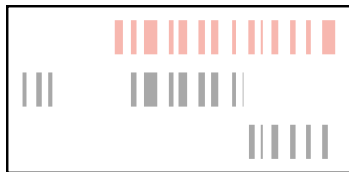

## Structural Category

- Fusion
- Reference Transcripts

0.087

0.0868

0.0866

0.0864

Chr X Position (Mb)

Supplement: S1 Fig — (PDF) [file pntd.0008869.s001.pdf]

GO Term

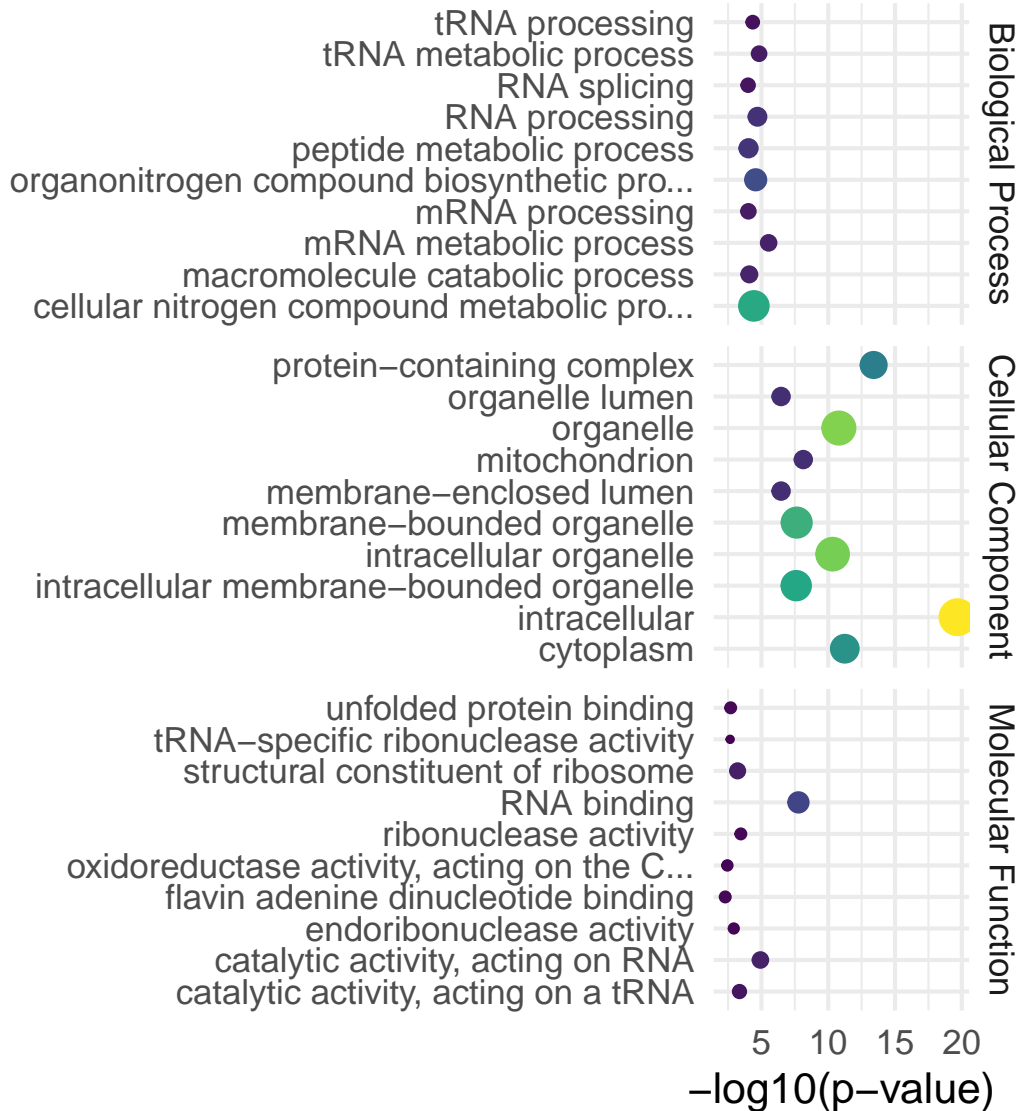

Annotated Genes

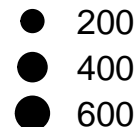

Annotated Genes

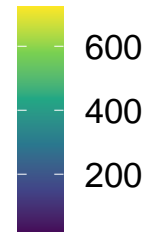

Supplement: S2 Fig — (PDF) [file pntd.0008869.s002.pdf]

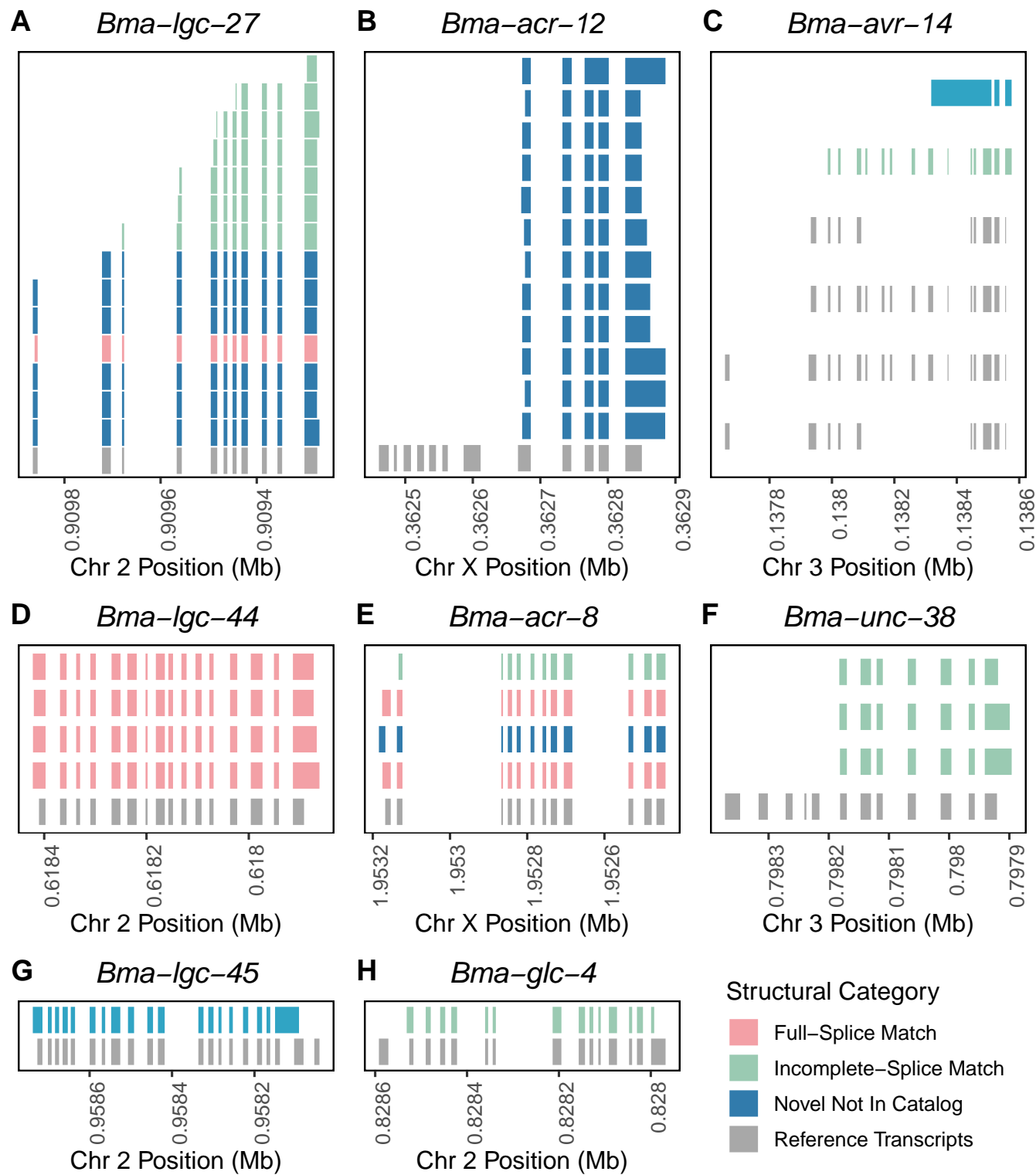

Supplement: S4 Fig — (PDF) [file pntd.0008869.s004.pdf]
