# Supplementary material for: Long-read RNA sequencing of human and animal filarial parasites improves gene models and discovers operons
Source: PLoS Negl Trop Dis. 2020 Nov 16;14(11):e0008869. doi: 10.1371/journal.pntd.0008869 (PMC7704054; doi:10.1371/journal.pntd.0008869)
Supplement: S3 Fig — (PDF) [file pntd.0008869.s003.pdf]

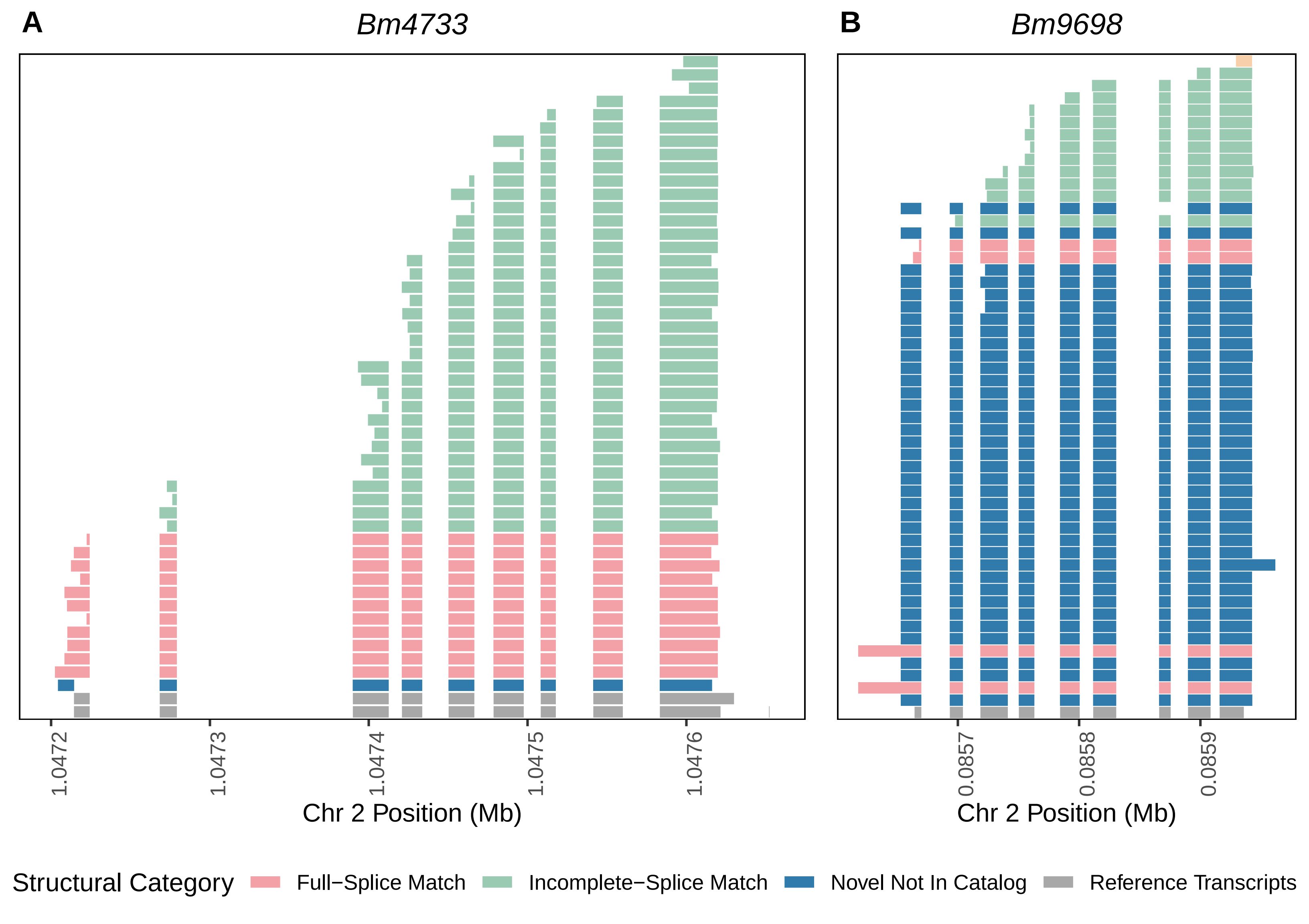
A
Bm4733
B
Bm9698
1.0472
1.0473
1.0474
1.0475
1.0476
0.0857
0.0858
0.0859
Chr 2 Position (Mb)
Chr 2 Position (Mb)
Structural Category
Full−Splice Match
Incomplete−Splice Match
Novel Not In Catalog
Reference Transcripts
