# Supplementary material for: Long-read RNA sequencing of human and animal filarial parasites improves gene models and discovers operons
Source: PLoS Negl Trop Dis. 2020 Nov 16;14(11):e0008869. doi: 10.1371/journal.pntd.0008869 (PMC7704054; doi:10.1371/journal.pntd.0008869)
Supplement: S5 Fig — (PDF) [file pntd.0008869.s005.pdf]

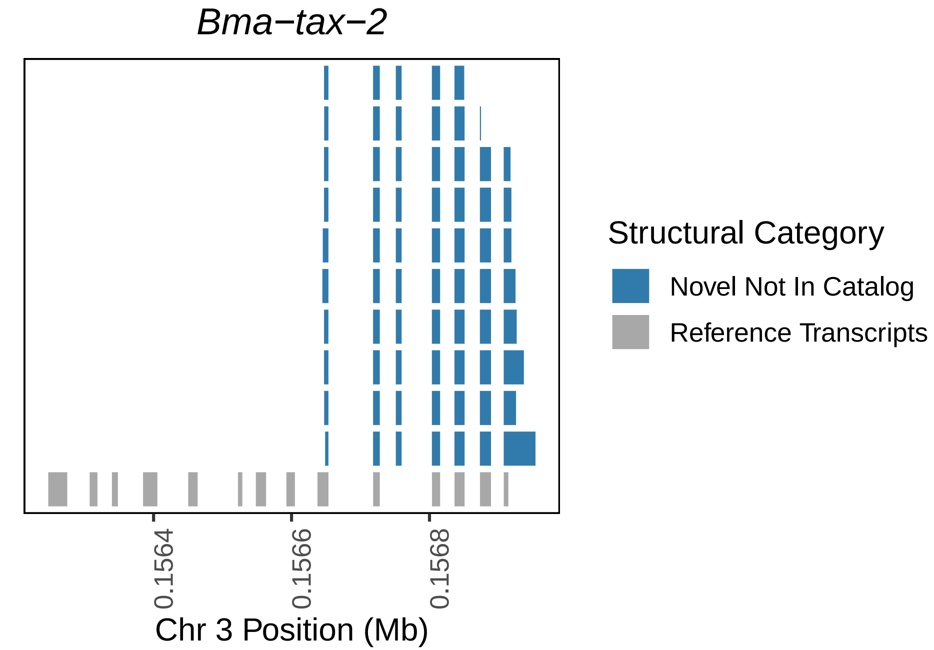
Bma−tax−2
Structural Category
Novel Not In Catalog
Reference Transcripts
0.1564
0.1566
0.1568
Chr 3 Position (Mb)

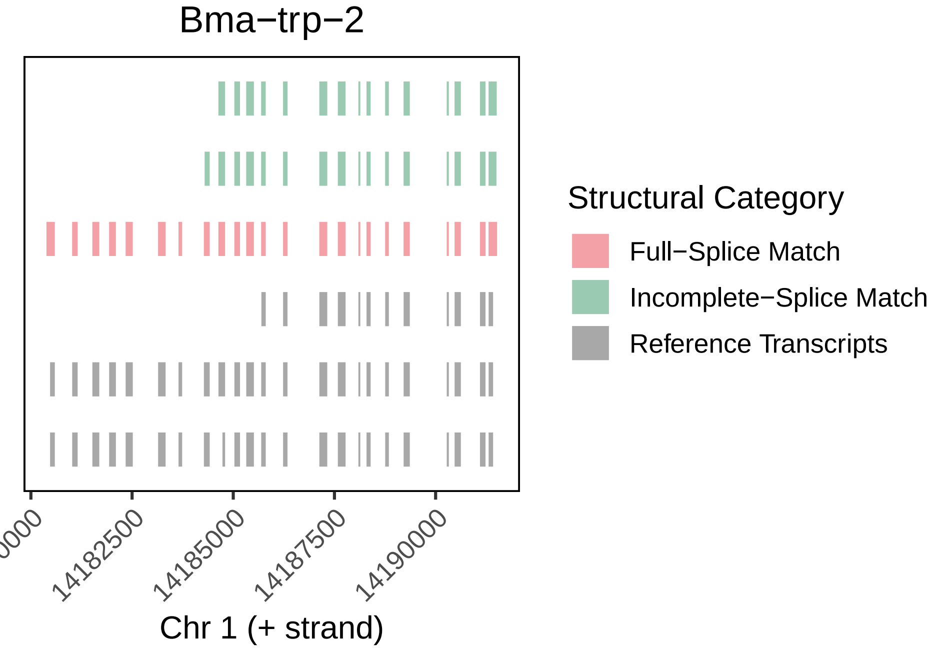
Bma−trp−2
Structural Category
Full−Splice Match
Incomplete−Splice Match
Reference Transcripts
14182500
14185000
14187500
14190000
Chr 1 (+ strand)
