# Supplementary material for: Long-read RNA sequencing of human and animal filarial parasites improves gene models and discovers operons
Source: PLoS Negl Trop Dis. 2020 Nov 16;14(11):e0008869. doi: 10.1371/journal.pntd.0008869 (PMC7704054; doi:10.1371/journal.pntd.0008869)
Supplement: S6 Fig — (PDF) [file pntd.0008869.s006.pdf]

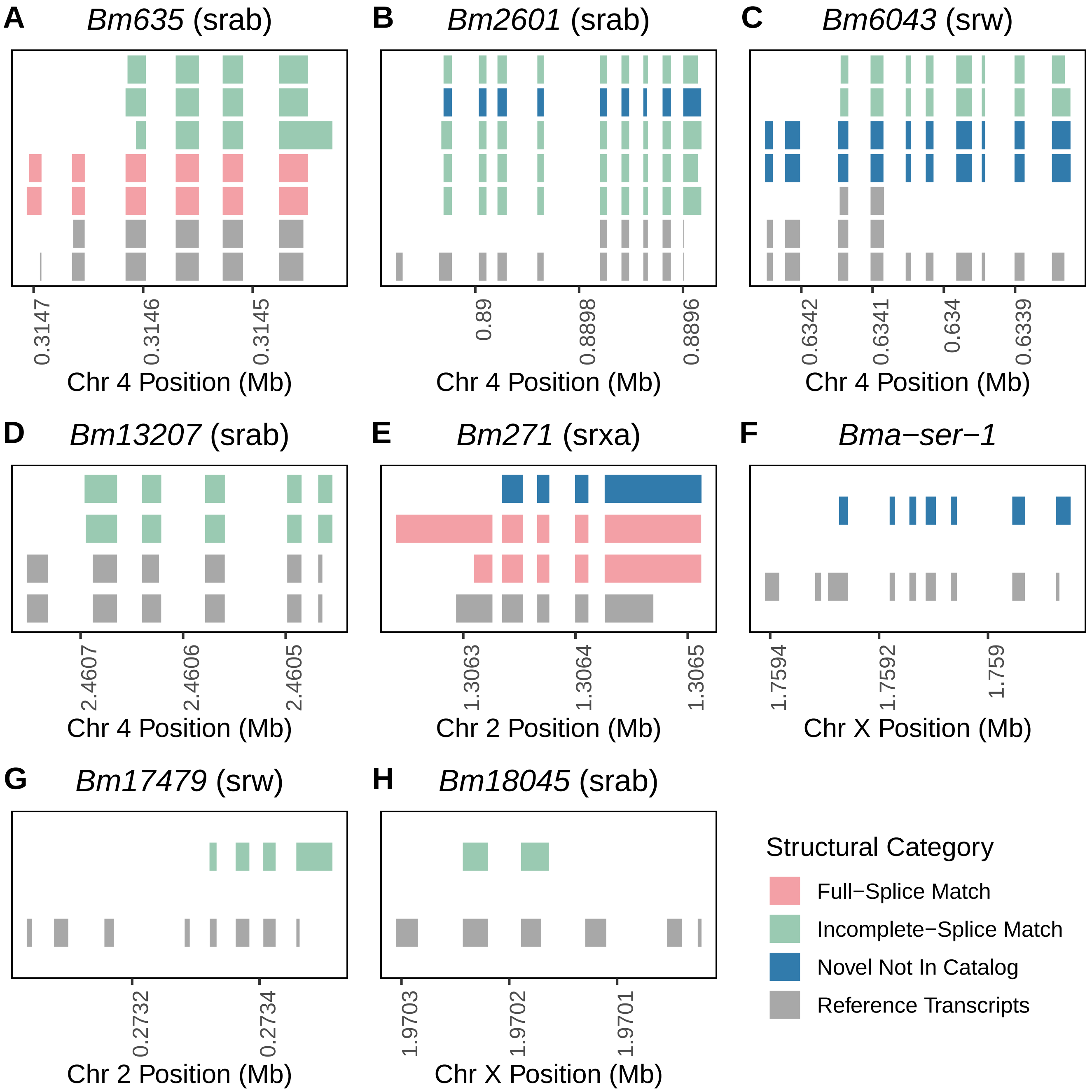
A
Bm635 (srab)
0.3147
0.3146
0.3145
Chr 4 Position (Mb)
B
Bm2601 (srab)
0.89
0.8898
0.8896
Chr 4 Position (Mb)
C
Bm6043 (srw)
0.6342
0.6341
0.634
0.6339
Chr 4 Position (Mb)
D
Bm13207 (srab)
2.4607
2.4606
2.4605
Chr 4 Position (Mb)
E
Bm271 (srxa)
1.3063
1.3064
1.3065
Chr 2 Position (Mb)
F
Bma−ser−1
1.7594
1.7592
1.759
Chr X Position (Mb)
G
Bm17479 (srw)
0.2732
0.2734
Chr 2 Position (Mb)
H
Bm18045 (srab)
1.9703
1.9702
1.9701
Chr X Position (Mb)
Structural Category
Full−Splice Match
Incomplete−Splice Match
Novel Not In Catalog
Reference Transcripts
